# Association of Serum Melatonin Level with Mild Cognitive Impairment in Type 2 Diabetic Patients: A Cross-Sectional Study

**DOI:** 10.1155/2021/5566019

**Published:** 2021-04-28

**Authors:** Jichen Zhang, Jiancan Lu, Hongling Zhu, Xinglu Zhou, Xijuan Wei, Mingjun Gu

**Affiliations:** ^1^The Graduate School, Ningxia Medical University, Yinchuan, Ningxia 750005, China; ^2^Department of Endocrinology, Shanghai Pudong New District Gongli Hospital, Second Military Medical University, Shanghai 200135, China

## Abstract

**Objectives:**

Melatonin is an essential neuroendocrine hormone that participates in the regulation of sleep rhythm and cognitive function. This study aimed to determine serum melatonin levels with mild cognitive impairment (MCI) in patients with type 2 diabetes (T2DM).

**Methods:**

A total of 247 T2DM patients were recruited in this retrospective study and divided into 75 subjects with MCI and 172 with normal cognition. Cognitive function was evaluated by the Montreal Cognitive Assessment (MoCA). Their blood sample was examined for the level of melatonin and other biochemical parameters.

**Results:**

Melatonin concentration was decreased in MCI patients to non-MCI patients (*P* < 0.001). Melatonin level was negatively correlated with age (*r* = −0.202; *P* = 0.001), diabetes duration (*r* = −0.282; *P* < 0.001), serum HbA1c (*r* = −0.195; *P* = 0.002), hs-CRP (*r* = −0.324; *P* < 0.001), and TSH (*r* = −0.184; *P* = 0.004) levels and positively correlated with MoCA score, serum HDL-C (*r* = 0.145; *P* < 0.001), FT3 (*r* = 0.241; *P* < 0.001), and FT4 (*r* = 0.169; *P* = 0.008) levels. The multivariable analysis indicated that fewer years of formal education, longer diabetes duration, higher serum HbA1c, higher serum hs-CRP, and lower serum melatonin are the predisposing factors for MCI.

**Conclusion:**

Lower melatonin level was associated with cognitive impairment in patients with T2DM. Melatonin might serve as a potential protective molecule against cognitive dysfunction in T2DM.

## 1. Introduction

Diabetes mellitus (DM) is one common metabolic disorder with high levels of blood glucose that is caused by insufficient insulin secretion from *β*-cells (type 1 DM) or body resistance to insulin (type 2 DM). Diabetes is amalgamated with an increased risk of dementia and Alzheimer's disease (AD) [1]. Mild cognitive impairment (MCI) is a transitional phase between normal aging and dementia, with manifestations of a gradual loss of memory and executive function [2]. In diabetes-related cognitive impairment, there are several structural changes in the central nervous system (CNS), such as reduced hippocampal size and neurogenesis, brain tissue atrophy, and abnormal neural electrical properties changes [3–5]. Therefore, it is urgently needed to explore early diagnostic biomarkers for MCI in diabetic patients to retard cognitive deterioration and decrease the incidence of dementia in diabetes.

Melatonin is an indole neuroendocrine hormone that is produced and secreted by the pineal gland. It mainly regulates the human body's circadian biological rhythm and keeps the normal “sleep -awakening cycle.” Melatonin can regulate and affect the function of multiple organs, effectively control the immune system's function, and play an antistress role [6]. Studies have shown the relationships between melatonin and cognitive functions. Serum melatonin levels were significantly declined in Alzheimer's disease patients with cognitive impairment and older postoperative delirium patients undergoing major abdominal surgery [7, 8]. Besides, a potential role for melatonin in the relationship between circadian regulation of insulin secretion by the pancreatic islets and diabetes has been suggested [9]. Serum melatonin levels were significantly lower in patients of both type 1 and type 2 diabetes [10, 11]. Therefore, melatonin may be involved in the genesis of diabetes as it induces a phase shift in insulin secretion. In contrast, dysregulation of circadian insulin secretion is an essential feature of type 2 diabetes [12]. Thus, melatonin has been suggested as a therapeutic target for type 2 diabetes, and this was further verified by the protective effects of exogenous melatonin in diabetes-induced neurobehavioral changes [13, 14]. Thus, melatonin probably plays a previously unrecognized role in T2DM-related cognitive impairment. However, it remains unclear about the relationship between melatonin and cognitive function in diabetic patients. Therefore, we hypothesized that melatonin might influence the susceptibility to early cognitive dysfunction in T2DM patients.

This study is aimed to explore the potential link between serum melatonin levels and cognitive function in diabetic patients.

## 2. Materials and Methods

### 2.1. Study Population

This study was conducted in the Department of Endocrinology of Shanghai Pudong New District Gongli Hospital from January 2017 to December 2019. The informed consent was obtained from participants or nearest relatives, and the Research Ethics Committee approved the study protocol of Gongli Hospital on November 22, 2016. A total of 247 hospitalized T2DM patients were recruited in this study, with a diabetes history >3 years. All diabetic patients were diagnosed according to the World Health Organization 1999 [15]. Cognitive function criteria were evaluated by the Montreal Cognitive Assessment (MoCA), and MCI was diagnosed based on the measures proposed by the MCI Working Group of the European Consortium [16]. The excluded subjects were as follows: (1) Presence of diabetic complications, such as severe hypoglycemia, diabetic ketoacidosis, or diabetic coma. (2) Presence of diseases with cognitive dysfunction, such as stroke, head injury, dementia, Parkinson's disease, epilepsy, or other mental illnesses. (3) Major medical illness (severe heart failure, cancer, anemia, and severe infection) or difficult communication conditions. (4) Use of cognition-impairing drugs.

### 2.2. Critical Issues

Our study has raised the following unclear issues: the median age was significantly different between the studied groups (MCI around 62 years and without MCI 56 years), and this could account for part of the differences found in our results.

### 2.3. Clinical and Laboratory Data Collection

Demographic and clinical data were collected as age, gender, education years, medical history, body mass index (BMI), smoking, and drinking. Fasting blood samples were collected in the second morning after admission and were immediately centrifuged to separate serum, frozen at −80°C until analysis. Fasting blood was also collected from 150 healthy subjects with matched age and gender and served as a control group. Serum levels of fasting blood glucose (FBG), glycosylated hemoglobin (HbA1c), triglyceride (TG), total cholesterol (TC), low-density lipoprotein cholesterol (LDL-C), high-density lipoprotein cholesterol (HDL-C), creatinine, and high sensitivity C-reactive protein (hs-CRP) were measured using routine laboratory methods. All samples were analyzed in triplicate. Radioimmunoassay was applied to measure serum FT3, FT4, and TSH levels.

### 2.4. Cognitive Function Assessment

The Montreal Cognitive Assessment (MoCA) is a compassionate tool to assess the overall cognitive function and detect MCI. Total scores range from 0 to 30, and lower scores indicate poor cognitive function. In the present study, we applied the MoCA score to evaluate all diabetic patients' cognitive function. Diabetic patients were divided into MCI (MoCA < 26) and non-MCI groups (MoCA ≥ 26).

### 2.5. Serum Melatonin Level

Blood samples were collected from diabetic patients and healthy controls and were centrifuged to separate serum. Serum melatonin level (Cat No. ab213978; Abcam, UK) was determined by enzyme-linked immunosorbent assay (ELISA) kits.

### 2.6. Statistical Analysis

Data were expressed as median (interquartile range) for quantitative variables or number (percentage) for categorical variables. All statistical analysis was performed by SPSS Software version 20.0 (SPSS Inc., Chicago, Illinois, USA). The Mann–Whitney *U* test analyzed quantitative data, and categorical data were analyzed by *χ*^2^ test. Spearman correlation was performed to investigate the correlations of MoCA score or serum melatonin with clinical indicators. Logistic multivariate regression was carried out to determine the independent risk factors of cognitive impairment. *P* < 0.05 was considered as the criteria of statistical significance.

## 3. Results

### 3.1. General Description of T2DM Patients with and without MCI

The demographic and clinical parameters of diabetic patients are presented in Table 1. The *χ*^2^ test results showed no significant differences between the MCI and non-MCI groups about gender, smoking, drinking, and presence of hypertension. Furthermore, the Mann–Whitney *U* test showed that patients with MCI were older, less educated, had higher BMI, longer duration of diabetes, higher serum levels of HbA1c (%), TG, TC, LDL-C, hs-CRP, and TSH, and lower level of HDL-C, FT3, and FT4 (Table 1). MoCA score was significantly lower in the MCI group compared with the non-MCI group. Lastly, no significant differences were found between the groups in levels of fasting blood glucose (FBG) and creatinine (*P* > 0.05).

### 3.2. The Correlations of MoCA Score with Clinical Indicators in T2DM Patients

Spearman's correlation analysis was accomplished to examine the associations of cognitive function with clinical indicators. The MoCA scores were negatively correlated with age (*r* = −0.202; *P* = 0.001), diabetes duration (*r* = −0.282; *P* < 0.001), HbA1c (*r* = −0.195; *P* = 0.002), TG (*r* = −0.137; *P* = 0.031), LDL-C (*r* = −0.132; *P* = 0.038), hs-CRP (*r* = −0.324; *P* < 0.001), TSH (*r* = −0.184; *P* = 0.004), whereas positively correlated with education years (*r* = 0.150; *P* = 0.019), HDL-C (*r* = 0.145; *P* < 0.001), FT3 (*r* = 0.241; *P* < 0.001) and FT4 (*r* = 0.169; *P* = 0.008) (Table 2). No correlations of MoCA score with FBG, TC, or creatinine were found (all *P* > 0.05).

### 3.3. Serum Melatonin Level and Risk of MCI

**Figure 1:**
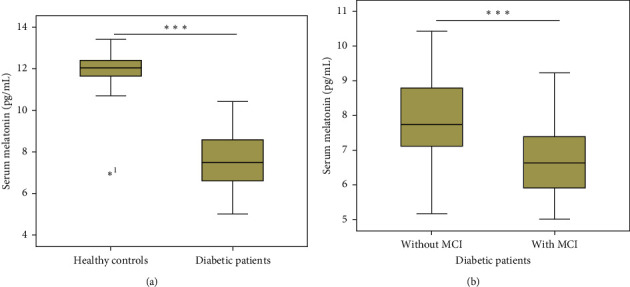
Serum levels of melatonin in diabetic patients with and without MCI. All data are expressed as medians and interquartile ranges (IQR). Mann–Whitney *U* tests were performed to compare the differences between groups. MCI: mild cognitive impairment.

The Mann–Whitney *U* test showed that the serum levels of melatonin were significantly lower in T2DM patients [7.5 pg/mL (IQR 6.62–8.56] compared to healthy controls [12.04 pg/mL (IQR 11.65–12.43] (*P* < 0.001; Figure 1(a)). Among all T2DM patients, serum levels of melatonin were significantly lower in the MCI group [6.66 pg/mL (IQR 5.87–7.4] than in the non-MCI group [7.73 pg/mL (IQR 7.1–8.8] (*P* < 0.001; Figure 1(b)).

### 3.4. The Correlations of Serum Melatonin Level with Other Clinical Indicators in T2DM Patients

**Figure 2:**
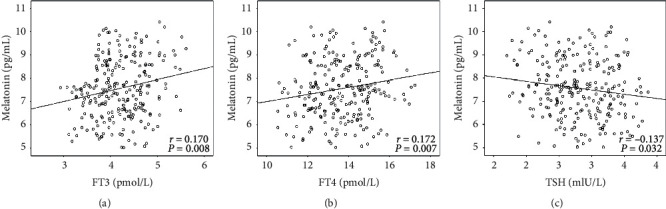
Correlation of serum melatonin to thyroid hormone in diabetic patients. Serum melatonin levels are positively correlated with (a) FT3 (*r* = 0.170, *P* = 0.008) and (b) FT4 (*r* = 0.172, *P* = 0.007) and are negatively correlated with (c) TSH (*r* = −0.137, *P* = 0.032). Spearman's rank correlation test was performed.

Spearman's correlation showed that serum melatonin levels were negatively correlated with age (*r* = −0.159, *P* = 0.012), BMI, diabetes duration, HbA1c, hs-CRP, whereas positively correlated with HDL-C (Table 3). Furthermore, positive correlations between serum melatonin level and MoCA test scores were observed. There were no significant correlations of serum melatonin with education year, FBG, TG, TC, LDL-C, or creatinine (*P* > 0.05). Spearman's correlation analysis was also performed to explore the associations of serum melatonin with thyroid hormone levels. The serum melatonin protein in diabetic patients correlated positively serum FT3 (*r* = 0.170, *P* = 0.008; Figure 2(a)) and FT4 (*r* = 0.172, *P* = 0.007; Figure 2(b)), and correlated negatively with TSH (*r* = −0.137) (*P* = 0.032; Figure 2(c)).

### 3.5. Logistic Regression Models

**Figure 3:**
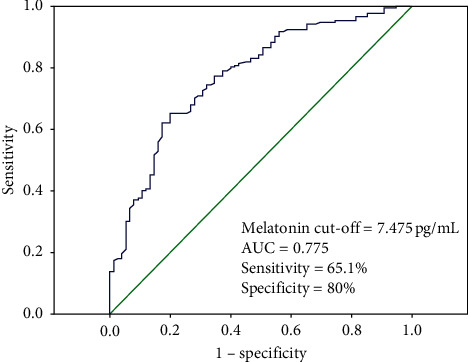
Receiver operator characteristic (ROC) curve of serum melatonin. The ROC curve was plotted to determine the cut-off point for serum melatonin that distinguishes the MCI and non-MCI in diabetic patients. MCI: mild cognitive impairment.

We performed multivariate logistic regressions to evaluate the risk factors of MCI in T2DM. The results showed that the independent risk factors associated with MCI in the T2DM patients included shorter education year (OR = 0.746, 95% CI = 0.616–0.903; *P* = 0.003), longer duration of T2DM (OR = 2.263, 95% CI = 1.496–3.424; *P* < 0.001), higher levels of HbA1c (OR = 3.641, 95% CI = 1.220–10.865; *P* = 0.0120), TG (OR = 2.343, 95% CI = 1.008–5.443; *P* = 0.048), hs-CRP (OR = 5.813, 95% CI = 2.074–16.295; *P* = 0.001), TSH (OR = 2.968, 95% CI = 1.260–6.992; *P* = 0.013) and lower level of FT3 (OR = 0.375, 95% CI = 0.169–0.835; *P* = 0.016) and melatonin (OR = 0.427, 95% CI = 0.305–9.599; *P* < 0.001) (all *P* < 0.05) (Table 4). Then receiver operating characteristic (ROC) curve was plotted. The optimal cut-off value of serum melatonin level to diagnose the MCI was 7.475 pg/mL, which yielded the highest sensitivity (65.1%) and specificity (80%; AUC = 0.775, 95% CI 0.712–0.838; *P* < 0.001; Figure 3). Patients with high serum melatonin (>7.475 pg/mL) had a higher risk of cognitive impairment (Adjusted OR = 0.191; 95% CI = 0.084–0.431) (Table 5).

## 4. Discussion

We carried out a cross-sectional investigation in our study to measure serum levels of melatonin and its association with MCI in T2DM patients. Our main findings were as follows (1) serum levels of melatonin were lower in T2DM patients with MCI compared to patients without MCI; (2) serum melatonin levels were negatively associated with age, BMI, diabetes duration, HbA1c, and hs-CRP; (3) reduced levels of melatonin were associated with an increased risk of MCI. These findings suggest that reduced melatonin levels may be related to the deterioration of cognition in diabetes.

In this study, low serum levels of melatonin were found in patients with T2DM and diabetic patients with MCI. Consistent with our findings, the serum levels of melatonin were decreased in type 1 and type 2 diabetic patients compared to healthy subjects [10, 11]. At the same time, another study showed that low serum melatonin was associated with autonomic neuropathy in type 2 diabetic patients and indicated that the circadian rhythm of melatonin secretion is blunted in type 2 diabetic patients [17]. Similarly, reduced melatonin was also associated with cognitive dysfunction in patients with Alzheimer's disease, schizophrenia, and even healthy older people [7, 18, 19]. However, it remains unclear about the association between serum melatonin and MCI in T2DM patients. This study showed that compared to diabetic patients with normal cognitive function, patients with MCI had significantly lower melatonin serum levels. Moreover, low melatonin level is an independent contributor to MCI. Our study provides serum melatonin as a biomarker of MCI, and reduced serum melatonin levels may be associated with cognitive deterioration.

We observed a negative correlation of serum melatonin level with serum HbA1c. The diabetes duration and HbA1c increased the risk of MCI in T2DM, and higher HbA1c was associated with cognitive impairment and lower executive function in older adults [20, 21]. These observations were confirmed by this study that patients with MCI had longer diabetes duration markedly and higher serum HbA1c levels compared to patients without MCI. The negative correlation between serum melatonin and diabetes duration also suggests that deregulation of melatonin secretion occurs in the early phase of type 2 diabetes. Therefore, melatonin might act as a molecule that retard the progression of MCI. Whether serum melatonin is changed in the prediabetic stage of T2DM is unclear and deserves further investigation. Our study also showed a negative correlation between serum melatonin level with age. Aging is an independent risk factor and contributor to cognitive dysfunction, with a significantly higher prevalence of MCI in subjects older than 70 years [22]. Melatonin is also associated with aging, and serum melatonin levels were gradually declined with aging [23]. As an antiaging protein, melatonin ameliorates aging-induced changes, including aging-induced cognitive impairment [24]. The autoregulation between aging and MCI in T2DM under the condition of low serum melatonin is unclear and deserves further study.

We have also investigated that serum melatonin levels were negatively correlated with hs-CRP level. Diabetes is a series of diseases whose pathological processes are associated with chronic inflammatory responses. In fact, hyperglycemia can activate one important inflammatory signal pathway NFκB and result in diabetic complications, including diabetic neuropathy and cognitive impairment [25]. NFκB is involved in pathological brain inflammation and is associated with the expression of proinflammatory cytokines [26]. These proinflammatory markers are elevated in patients with type 2 DM, including C-reactive protein [27], neutrophil/lymphocyte ratio [28], platelet/lymphocyte ratio [29], uric acid/HDL ratio [30], and mean platelet volume [31]. Our study showed hs-CRP was higher in MCI diabetics compared to non-MCI diabetics. This indicates that serum hs-CRP is not merely a marker of inflammation of diabetes but also is associated with diabetic cognitive impairment [32]. Moreover, serum hs-CRP was negatively correlated with melatonin levels. Our data suggest that melatonin may have some function in decreasing inflammatory burden in type 2 DM. This observation is also consistent with previous investigations that melatonin supplementation significantly decreased serum levels of inflammatory mediators, including TNF-*α* and IL-6 [33]. This indicates melatonin is an anti-inflammatory protein and can potentially combat systematic inflammatory response in diabetes [34]. An experimental study showed that melatonin administration effectively prevented mild inflammation in high-fat diet-induced metabolic syndrome (MS) rats [35]. Through inhibition of neuroinflammation, melatonin can attenuate cognitive impairment induced by various pathological conditions [36, 37]. Besides, the anti-inflammatory action of melatonin might be associated with its antiobesity effect. In high-fat diet-induced mice, melatonin supplementation decreased the gene expression of inflammation-related factors and prevented mass body gain [38]. Our results support that serum melatonin showed a negative correlation with the BMI of diabetic patients. Thus, melatonin can serve as a potential therapeutic agent to improve MCI through attenuation of inflammatory responses [39].

We showed the associations of serum melatonin with thyroid hormone levels in diabetic patients. Thyroid hormone level is closely related to the occurrence of T2DM. It affects the proliferation of pancreatic beta cells and insulin secretion and changes the sensitivity of liver and adipose tissue to insulin, and has the effect of increasing blood glucose. T2DM patients showed a significantly higher prevalence of thyroid dysfunction and serum TSH and T3 levels [40]. Furthermore, thyroid hormone is associated with psychiatric symptoms and cognitive impairment. T3 is the active form of thyroid hormone and could regulate neural stem cell function in the hippocampus and involve the adult mammalian brain's neurogenesis [41]. Primary hypothyroidism is a hypometabolic syndrome caused by thyroid diseases. Adult-onset hypothyroidism may cause cognitive impairment in memory, reaction ability, attention, and executive function [42]. When thyroid hormone changes in the normal range, it has a particular impact on cognitive function. For instance, even in subjects with normal thyroid function, the thyroid hormone FT4 level was positively correlated with cognitive function [43]. However, it remains unclear about the relationship between thyroid hormone and cognitive impairment of diabetes. An experimental study showed that thyroid hormone T3 treatment showed neuroprotective function in diabetic rats by reducing tau proteins' accumulation and activation of the neurodegenerative pathway in the hippocampus [44]. These results are consistent with our findings that diabetic patients with cognitive impairment showed lower serum FT3 and FT4 than patients with normal cognition. Therefore, our study adds thyroid hormone as a biomarker for cognitive impairment in diabetic patients. Whether changes in thyroid hormone in the early phase of diabetes indicate later cognitive impairment is an interesting question with important clinical significance and deserves further longitudinal study.

The positive correlations of melatonin with FT3 and FT4 indicate that thyroid hormone might mediate melatonin's protection on cognitive impairment of diabetes. Melatonin has been found to have a promotive effect on thyroid hormone production. Melatonin treatment alone or combined with TSH increased the production of thyroid T4 in rats [45]. Though melatonin is primarily secreted by the pineal gland, and thyroid C-cells can also synthesize it to modulate thyroid activity through a paracrine way [46]. Thus, melatonin can be used as a potential hormone against hypothyroidism, and exogenous melatonin administration could attenuate the signs of hypothyroidism and increase the serum T3 and T4 levels [47]. Moreover, melatonin might also be involved in the process of cognitive impairment of hypothyroidism, as melatonin treatment reversed the decline in serum thyroid hormone and reduced the neural apoptosis in newborn rats induced by maternal hypothyroidism [48]. Whether thyroid hormone involves melatonin's protective effect on diabetic cognitive impairment is unclear and deserves further investigation.

There were several limitations to this study. Firstly, as we applied the MoCA as the only assessment tool to evaluate the participants' cognitive cognition, the more cognitive score should be used to obtain more accurate results. Secondly, the relationships between melatonin and cognitive function were studied among a T2DM population. A more detailed study should be carried out in T2DM patients with MCI and no MCI. Thirdly, this is a cross-sectional study, and a longitudinal investigation is required to study the change of melatonin in the early phase of type 2 diabetes.

In conclusion, melatonin concentration is decreased in the MCI diabetic patients and is positively associated with cognitive function and negatively correlated with age, BMI, diabetes duration, HbA1c, and hs-CRP. The serum melatonin level might be a biomarker of cognitive function and become a strong predictor of MCI in patients with type 2 diabetes mellitus.

## Figures and Tables

**Table 1:** Demographic and clinical characteristics of study population.

Variables	Non-MCI (*n* = 172)	MCI (*n* = 75)	*Z* or *χ*^2^	*P* value
Age (year)	56.5 (53–64)	62 (56–67)	−3.081	0.002
Male (*n* (%))	93 (54.1%)	36 (48%)	0.771	0.380
Education (years)	12 (9–12)	9 (9–12)	−3.333	0.001
BMI	25.8 (24.6–26.7)	26.4 (25.6–27.3)	−2.423	0.015
Diabetes duration (years)	8.13 (7.55–8.72)	8.97 (8.28–9.74)	−5.971	<0.001
Smoking	48 (27.9%)	27 (36%)	1.618	0.203
Drinking	38 (22.1%)	22 (29.3%)	1.489	0.222
Hypertension	68 (39.5%)	35 (46.7%)	1.093	0.296
FBG (mmol/L)	8.91 (8.64–9.15)	8.9 (8.67–9.16)	−0.389	0.697
HbA1c (%)	9.45 (9.19–9.63)	9.59 (9.4–9.75)	−3.551	<0.001
TG (mmol/L)	2.55 (2.21–2.85)	2.71 (2.42–2.98)	−2.560	0.010
TC (mmol/L)	6.04 (5.78–6.35)	6.24 (5.88–6.49)	−2.199	0.028
LDL-C (mmol/L)	3.53 (3.26–3.78)	3.76 (3.32–3.97)	−2.699	0.007
HDL-C (mmol/L)	1.74 (1.54–2.03)	1.67 (1.46–1.82)	−2.458	0.014
Creatinine (*μ*mol/L)	79.1 (71.3–84.1)	80.2 (76–84.3)	−1.560	0.119
Hs-CRP (ng/mL)	2.1 (1.77–2.42)	2.45 (2.15–2.64)	−5.346	<0.001
FT3 (pmol/L)	4.24 (3.91–4.66)	3.97 (3.71–4.30)	−2.577	<0.001
FT4 (pmol/L)	13.7 (12.9–14.9)	13.4 (12.2–14.4)	−2.309	0.017
TSH (mIU/L)	2.73 (2.33–3.14)	2.96 (2.60–3.26)	−3.193	0.001
MoCA	28 (27–29)	23 (22–24)	−12.597	<0.001

Abbreviations: T2DM: type 2 diabetes; BMI: body mass index; FBG: fasting blood glucose; HbAlc: glycosylated hemoglobin; TG: triglyceride; TC: total cholesterol; LDL-C: low-density lipoprotein cholesterol; HDL-C: high-density lipoprotein cholesterol; hs-CRP: high sensitivity C-reactive protein; MoCA: Montreal Cognitive Assessment. Data are expressed as medians and interquartile ranges (IQR) for quantitative variables and expressed as cases and percentages for category variables. Mann–Whitney *U* test (*Z*), or *χ*^2^ test was used to test for significant differences. FT3: serum-free triiodothyronine; FT4:serum-free thyroxine; TSH: thyroid stimulating hormone.

**Table 2:** The correlations of MoCA score with clinical indicators in T2DM patients.

MoCA		
Variables	*r*	*P* value
Age (year)	−0.202	0.001
Education (years)	0.150	0.019
BMI	−0.100	0.117
Diabetes duration (years)	−0.282	<0.001
FBG (mmol/L)	−0.022	0.732
HbA1c (%)	−0.195	0.002
TG (mmol/L)	−0.137	0.031
TC (mmol/L)	−0.081	0.207
LDL-C (mmol/L)	−0.132	0.038
HDL-C (mmol/L)	0.145	0.022
Creatinine (*μ*mol/L)	−0.041	0.525
Hs-CRP (ng/mL)	−0.324	<0.001
FT3 (pmol/L)	0.241	<0.001
FT4 (pmol/L)	0.169	0.008
TSH (mIU/L)	−0.184	0.004

Abbreviations: MoCA: Montreal Cognitive Assessment; BMI: body mass index; FBG: fasting blood glucose; HbAlc: glycosylated hemoglobin; TG: triglyceride; TC: total cholesterol; LDL-C: low-density lipoprotein cholesterol; HDL-C: high-density lipoprotein cholesterol; hs-CRP: high sensitivity C-reactive protein. Spearman correlation was performed.

**Table 3:** The correlations of serum melatonin level with other clinical indicators and cognitive performances in T2DM patients.

Variables	Melatonin	
	*r*	*P* value
Age (year)	−0.159	0.012
Education (years)	0.075	0.237
BMI	−0.140	0.027
Diabetes duration (years)	−0.183	0.004
FBG (mmol/L)	−0.072	0.260
HbA1c (%)	−0.148	0.020
TG (mmol/L)	−0.056	0.382
TC (mmol/L)	−0.025	0.691
LDL-C (mmol/L)	−0.106	0.097
HDL-C (mmol/L)	0.150	0.019
Creatinine (*μ*mol/L)	−0.075	0.239
Hs-CRP (ng/mL)	−0.234	<0.001
MoCA	0.353	<0.001

Abbreviations: BMI: body mass index; FBG: fasting blood glucose; HbAlc: glycosylated hemoglobin; TG: triglyceride; TC: total cholesterol; LDL-C: low-density lipoprotein cholesterol; HDL-C: high-density lipoprotein cholesterol; hs-CRP: high sensitivity C-reactive protein; MoCA: Montreal Cognitive Assessment. Spearman correlation was performed.

**Table 4:** Logistic multivariate regression evaluates the risk of MCI in T2DM patients.

Variables	*β*	SE of *β*	*P* value	OR	95% CI
Education (years)	−0.293	0.098	0.003	0.746	0.616–0.903
Diabetes duration (years)	0.817	0.211	<0.001	2.263	1.496–3.424
HbA1c (%)	1.292	0.558	0.020	3.641	1.220–10.865
TG (mmol/L)	0.851	0.430	0.048	2.343	1.008–5.443
Hs-CRP (ng/mL)	1.760	0.526	0.001	5.813	2.074–16.295
FT3 (pmol/L)	−0.980	0.408	0.016	0.375	0.169–0.835
TSH (mIU/L)	1.088	0.437	0.013	2.968	1.260–6.992
Melatonin (pg/mL)^*∗*^	−0.851	0.172	<0.001	0.427	0.305–9.599

Abbreviations: *β*: regression coefficient; SE: standard error; OR: odds ratio; CI: confidence interval for odds ratio; MCI: mild cognitive impairment; HbAlc: glycosylated hemoglobin; hs-CRP: high sensitivity C-reactive protein.

**Table 5:** The risk of cognitive impairment in T2DM patients with high serum melatonin.

Variable	OR (95% CI)	Adjusted OR (95% CI)
Serum melatonin	0.170 (0.069–0.423)	0.191 (0.084–0.431)

Abbreviations: OR: odds ratio; CI: confidence interval for odds ratio.

## Data Availability

The data used to support the findings of this study are available from the corresponding author upon request.

## References

[1] Ott A., Stolk R. P., van Harskamp F., Pols H. A. P., Hofman A., Breteler M. M. B. (1999). Diabetes mellitus and the risk of dementia: the Rotterdam study. *Neurology*.

[2] Whitmer R. A., Karter A. J., Yaffe K. (2009). Hypoglycemic episodes and risk of dementia in older patients with type 2 diabetes mellitus. *JAMA*.

[3] Ramos-Rodriguez J. J., Molina-Gil S., Ortiz-Barajas O. (2014). Central proliferation and neurogenesis is impaired in type 2 diabetes and prediabetes animal models. *PLoS One*.

[4] Wrighten S. A., Piroli G. G., Grillo C. A. (2009). A look inside the diabetic brain: contributors to diabetes-induced brain aging. *Biochimica et Biophysica Acta*.

[5] Beauquis J., Saravia F., Coulaud J. (2008). Prominently decreased hippocampal neurogenesis in a spontaneous model of type 1 diabetes, the nonobese diabetic mouse. *Experimental Neurology*.

[6] Opie L. H., Lecour S. (2016). Melatonin has multiorgan effects. *European Heart Journal-Cardiovascular Pharmacotherapy*.

[7] Sirin F. B., Kumbul Doğuç D., Vural H. (2015). Plasma 8-isoPGF2*α* and serum melatonin levels in patients with minimal cognitive impairment and Alzheimer disease. *Turkish Journal of Medical Sciences*.

[8] Shen Q. H., Li H. F., Zhou X. Y. (2020). Relation of serum melatonin levels to postoperative delirium in older patients undergoing major abdominal surgery. *Journal of International Medical Research*.

[9] Sharma S., Singh H., Ahmad N., Mishra P., Tiwari A. (2015). The role of melatonin in diabetes: therapeutic implications. *Archives of Endocrinology and Metabolism*.

[10] Kor Y., Geyikli I., Keskin M., Akan M. (2014). Preliminary study: evaluation of melatonin secretion in children and adolescents with type 1 diabetes mellitus. *Indian Journal of Endocrinology and Metabolism*.

[11] Peschke E., Frese T., Chankiewitz E. (2006). Diabetic Goto Kakizaki rats as well as type 2 diabetic patients show a decreased diurnal serum melatonin level and an increased pancreatic melatonin-receptor status. *Journal of Pineal Research*.

[12] Elmar P., Eckhard M. (2010). New evidence for a role of melatonin in glucose regulation. *Best Practice & Research Clinical Endocrinology & Metabolism*.

[13] Marzena W., Michal K., Pawel W. (2017). Melatonin as a pleiotropic molecule with therapeutic potential for type 2 diabetes and cancer. *Current Medicinal Chemistry*.

[14] Jangra A., Datusalia A. K., Khandwe S., Sharma S. S. (2013). Amelioration of diabetes-induced neurobehavioral and neurochemical changes by melatonin and nicotinamide: implication of oxidative stress-PARP pathway. *Pharmacology Biochemistry and Behavior*.

[15] Alberti K. G. M. M., Zimmet P. Z. (1998). Definition, diagnosis and classification of diabetes mellitus and its complications. part 1: diagnosis and classification of diabetes mellitus. provisional report of a WHO consultation. *Diabetic Medicine*.

[16] Portet F., Ousset P. J., Visser P. J. (2006). Mild cognitive impairment (MCI) in medical practice: a critical review of the concept and new diagnostic procedure. report of the MCI working group of the European consortium on Alzheimer’s disease. *Journal of Neurology, Neurosurgery & Psychiatry*.

[17] Tutuncu N. B., Batur M. K., Yildirir A. (2005). Melatonin levels decrease in type 2 diabetic patients with cardiac autonomic neuropathy. *Journal of Pineal Research*.

[18] Sahbaz C., Özer O. F., Kurtulmus A., Kırpınar I., Sahin F., Guloksuz S. (2019). Evidence for an association of serum melatonin concentrations with recognition and circadian preferences in patients with schizophrenia. *Metabolic Brain Disease*.

[19] Kurowska A., Bodys-Cupak I., Staszkiewicz M. (2020). Interleukin-6 and melatonin as predictors of cognitive, emotional and functional ageing of older people. *International Journal of Environmental Research and Public Health*.

[20] Gao Y., Xiao Y., Miao R. (2016). The prevalence of mild cognitive impairment with type 2 diabetes mellitus among elderly people in China: a cross-sectional study. *Archives of Gerontology and Geriatrics*.

[21] Pappas C., Small B. J., Andel R. (2019). Blood glucose levels may exacerbate executive function deficits in older adults with cognitive impairment. *Journal of Alzheimer’s Disease*.

[22] Petersen R. C., Roberts R. O., Knopman D. S. (2009). Mild cognitive impairment: ten years later. *Archives of Neurology*.

[23] Hill S., Cheng C., Yuan L. (2013). Age-related decline in melatonin and its MT1 receptor are associated with decreased sensitivity to melatonin and enhanced mammary tumor growth. *Current Aging Science*.

[24] Hosseini L., Farokhi-Sisakht F., Badalzadeh R., Khabbaz A., Mahmoudi J., Sadigh-Eteghad S. (2019). Nicotinamide mononucleotide and melatonin alleviate aging-induced cognitive impairment via modulation of mitochondrial function and apoptosis in the prefrontal cortex and hippocampus. *Neuroscience*.

[25] Muriach M., Flores-Bellver M., Romero F. J. (2014). Diabetes and the brain: oxidative stress, inflammation, and autophagy. *Oxidative Medicine and Cellular Longevity*.

[26] Chen L., Hu L., Zhao J. (2016). Chotosan improves A*β* 1–42-induced cognitive impairment and neuroinflammatory and apoptotic responses through the inhibition of TLR-4/NF-*κB* signaling in mice. *Journal of Ethnopharmacology*.

[27] Tekce H., Tekce B., Aktas G., Alcelik A., Sengul E. (2014). Serum omentin-1 levels in diabetic and nondiabetic patients with chronic kidney disease. *Experimental and Clinical Endocrinology & Diabetes*.

[28] Duman T. T., Aktas G., Atak B. M., Kocak M. Z., Erkus E., Savli H. (2019). Neutrophil to lymphocyte ratio as an indicative of diabetic control level in type 2 diabetes mellitus. *African Health Sciences*.

[29] Ilgun E., Akyurek O., Kalkan A. O., Demir F., Demirayak M., Bilgi M. (2016). Neutrophil/lymphocyte ratio and platelet/lymphocyte ratio in fibromyalgia. *Electronic Journal of General Medicine*.

[30] Aktas G., Kocak M. Z., Bilgin S., Atak B. M., Duman T. T., Kurtkulagi O. (2019). Uric acid to HDL cholesterol ratio is a strong predictor of diabetic control in men with type 2 diabetes mellitus. *The Aging Male*.

[31] Ulasli S. S., Ozyurek B. A., Yilmaz E. B., Ulubay G. (2012). Mean platelet volume as an inflammatory marker in acute exacerbation of chronic obstructive pulmonary disease. *Polish Archives of Internal Medicine*.

[32] Gorska-Ciebiada M., Ciebiada M. (2020). Association of hsCRP and vitamin D levels with mild cognitive impairment in elderly type 2 diabetic patients. *Experimental Gerontology*.

[33] Zarezadeh M., Khorshidi M., Emami M. (2020). Melatonin supplementation and pro-inflammatory mediators: a systematic review and meta-analysis of clinical trials. *European Journal of Nutrition*.

[34] Maher A. M., Saleh S. R., Elguindy N. M., Hashem H. M., Yacout G. A. (2020). Exogenous melatonin restrains neuroinflammation in high fat diet induced diabetic rats through attenuating indoleamine 2, 3-dioxygenase 1 expression. *Life Sciences*.

[35] Cano Barquilla P., Pagano E. S., Jiménez-Ortega V., Fernández-Mateos P., Esquifino A. I., Cardinali D. P. (2014). Melatonin normalizes clinical and biochemical parameters of mild inflammation in diet-induced metabolic syndrome in rats. *Journal of Pineal Research*.

[36] Yuan H., Wu G., Zhai X. (2019). Melatonin and rapamycin attenuate isoflurane-induced cognitive impairment through inhibition of neuroinflammation by suppressing the mTOR signaling in the hippocampus of aged mice. *Frontiers in Aging Neuroscience*.

[37] Yang B., Zhang L.-Y., Chen Y. (2020). Melatonin alleviates intestinal injury, neuroinflammation and cognitive dysfunction caused by intestinal ischemia/reperfusion. *International Immunopharmacology*.

[38] De Farias T. D. S. M., Cruz M. M., De Sa R. C. D. C. (2019). Melatonin supplementation decreases hypertrophic obesity and inflammation induced by high-fat diet in mice. *Frontiers in Endocrinology (Lausanne)*.

[39] Cardinali D. P., Hardeland R. (2017). Inflammaging, metabolic syndrome and melatonin: a call for treatment studies. *Neuroendocrinology*.

[40] Jali M. V., Kambar S., Jali S. M. (2017). Prevalence of thyroid dysfunction among type 2 diabetes mellitus patients. *Diabetes & Metabolic Syndrome*.

[41] Remaud S., Gothié J. D., Morvan-Dubois G. (2014). Thyroid hormone signaling and adult neurogenesis in mammals. *Frontiers in Endocrinology (Lausanne)*.

[42] Elgazar E. H., Esheba N. E., Shalaby S. A., Mohamed W. F. (2019). Thyroid dysfunction prevalence and relation to glycemic control in patients with type 2 diabetes mellitus. *Diabetes & Metabolic Syndrome: Clinical Research & Reviews*.

[43] Choi B. W., Kim S., Kang S., Won K. S., Yi H.-A., Kim H. W. (2020). Relationship between thyroid hormone levels and the pathology of Alzheimer’s disease in euthyroid subjects. *Thyroid*.

[44] Prieto-Almeida F., Panveloski-Costa A. C., Crunfli F., Da Silva Teixeira S., Nunes M. T., Torrão A. D. S. (2018). Thyroid hormone improves insulin signaling and reduces the activation of neurodegenerative pathway in the hippocampus of diabetic adult male rats. *Life Sciences*.

[45] Csaba G., Nagy S. U., Rom-Bugoslavskaya E. S., Shcherbakova V. S (1987). Effect of single neonatal melatonin treatment on in vitro thyroxin secretion and TSH or melatonin-modified cAMP level of one-month-old rats. *Acta Physiologica Hungarica*.

[46] Garcia-Marin R., Fernandez-Santos J. M., Morillo-Bernal J. (2015). Melatonin in the thyroid gland: regulation by thyroid-stimulating hormone and role in thyroglobulin gene expression. *Journal of Physiology and Pharmacology*.

[47] Albuquerque Y. M. L., Silva W. E. D., Souza F. A. L., Teixeira V. W, Teixeira ÁA. C (2020). Melatonin on hypothyroidism and gonadal development in rats: a review. *JBRA Assisted Reproduction*.

[48] Hidayat M., Chaudhry S., Salman S. (2019). Melatonin prevents apoptosis in brains of neonates induced by maternal hypothyroidism. *Journal of Ayub Medical College Abbottabad*.

